# P02.118. Oral chamomile (Matricaria recutita) extract therapy of Generalized Anxiety Disorder (GAD): trial in progress

**DOI:** 10.1186/1472-6882-12-S1-P174

**Published:** 2012-06-12

**Authors:** J Mao, R Derubeis, Q Li, J Baier, D Cristancho, J Hollm, J Minkel, J Amsterdam

**Affiliations:** 1 Perelman School of Medicine at University of Pennsylvania, Philadelphia, USA

## Purpose

Chamomile is a traditional herb known for its calming effects. We are conducting a long-term, randomized, placebo-substitution study of chamomile for the prevention of recurrent Generalized Anxiety Disorder (GAD) in individuals who have responded to initial, open-label chamomile therapy. We present preliminary results from the trial’s open label phase to offer initial evidence of safety and effectiveness of chamomile in GAD.

## Methods

A 38-week long-term relapse prevention trial with three phases. I: Eight weeks to determine whether individuals respond to chamomile; II: Four more weeks among responders to determine whether symptoms remain stable; III: An additional 26 weeks to determine whether chamomile is superior to placebo in preventing the recurrence of anxiety symptoms in responders. Subjects meet DSM IV-TR criteria for GAD, moderate severity. Study interventions include pharmaceutical grade chamomile extract (SHR-5) 1,500 mg daily standardized to 1.2% apigenin and comparable placebo. Primary outcome measures are the GAD-7, Hamilton Anxiety Rating (HAM-A), and Clinical Global Impression Severity (CGI/S) scales.

## Results

To date, 63 subjects have been enrolled, median age 48, range (24 to 71); 40 women, 23 men; 49 White, 6 African American, 6 Asian, 2 Other. Among the 48 subjects who completed phase I, a significant mean reduction of anxiety symptoms, as measured by GAD-7 (13.4 to 5.4, 59.7%, p≤0.001) and HAM-A (16.3 to 5.5, 66.2%, p≤0.001) have been observed. By *a priori* defined CGI-S and by 50% symptom reduction in GAD-7, 38 of those completing phase I (79.2%) met criteria for response. No serious adverse events were observed.

## Conclusion

Preliminary findings are consistent with our previous RCT finding that over 50% of subjects with moderate or severe GAD symptoms respond to chamomile. The 1500mg dose of chamomile oral extract appears to be safe. More definitive and long-term relapse prevention findings await the completion of the trial.

